# Influence of *Sesamia nonagrioides* and *Fusarium verticillioides* on carbon elemental and isotopic composition in maize stem piths

**DOI:** 10.1186/s13104-025-07278-0

**Published:** 2025-05-16

**Authors:** Elisa Fernández-Descalzo, Lorena Álvarez-Iglesias, Ana Butrón, Serafín J. González-Prieto

**Affiliations:** 1 Misión Biológica de Galicia, Sede de Santiago (CSIC), PO Box 122, Santiago de Compostela, E-15780 Spain; 2 Misión Biológica de Galicia, Sede de Pontevedra (CSIC), Pazo de Salcedo. Carballeira, 8, Salcedo, Pontevedra, E-36143 Spain

**Keywords:** Carbon, Resistance, Insect, Fungus, Interaction, Maize

## Abstract

The stem borer *Sesamia nonagrioides* and the fungus *Fusarium verticillioides* are frequently present in maize. However, their interaction with this crop and their effects on its physiology remain poorly understood. This study explores the combined impact of these two organisms on carbon composition and δ¹³C content in the stem pith. Eight genetically distinct maize lines (A239, A509, A630, A637, EP42, EP77, EP125, PB130) were subjected to four treatments: an untreated control (UC), infection by *F. verticillioides* (FV), attack by *S. nonagrioides* (SN), and infection by *F. verticillioides* and infestation by *S. nonagrioides* (FS). Results show moderate variation in δ¹³C values between UC genotypes (-12.6 to -13.1‰, with the exception of line EP77 at -13.8‰), with minor differences related to their inbred resistance to the insect and fungus. Insect infestation results in a noticeable reduction in δ¹³C (0.6‰), but this decrease is partially attenuated in plants previously colonized by the fungus, suggesting an influence of the fungus on the maize’s defensive response. In addition, the analysis of ^13^C isotopic composition and carbon signatures provide key elements for a better understanding of maize defence mechanisms. To our knowledge, this study is the first to explore the impact of *Sesamia* and *Fusarium*, and their interaction, on carbon metabolism in different maize genotypes.

## Highlights

Synergistic and/or antagonistic effects of *Fusarium* in maize infested by *Sesamia* are unknown.

The %C and δ ^13^C of maize were analysed in eight genotypes showed contrasting behaviours towards these two biotic agents.

The δ ^13^C of maize was slightly affected by inbred resistance to *Sesamia* and *Fusarium.*

Compared to control plants, δ ^13^C decreased by about 0.6‰ in plants infested by *Sesamia* and by about 0.3‰ in those infected by *Fusarium.*

The ^13^C isotopic and C content signatures provides relevant information about maize defence mechanisms.

## Introduction

With its production growing three times faster than that of wheat or rice in the 2000–2022 period, maize (*Zea mays* L.) is currently the second most produced crop worldwide after sugarcane [1].

Lepidoptera caterpillars that feed on maize stalk (‘stem borers’) are one of the most damaging insect pests of the crop [2]; among them, several *Sesamia* species are of particular concern around the world: *S. calamistis* Hampson, 1910, in Africa [3], *S. inferens* (Walker, 1856) in Asia [4] and *S. nonagrioides* (Lefèbvre, 1827) -thereafter *Sesamia*- in the Mediterranean area [5].

The fungus *Fusarium verticillioides* (Sacc.) Niremberg, 1976 -thereafter *Fusarium*- is often found inside maize kernels in temperate areas around the world [6] and poses a threat not only to crop yield, but also to animal and human health [7]. However, this fungus is also able of asymptomatic colonization as an endophyte and it can enhance host fitness at seedling stage by promoting growth and inducing defence-associated responses such as lignin deposition, and, at mature stage, it may also contribute to increased biomass [8–10].

The maize response to *Sesamia* could be mediated by the presence of *Fusarium* as seed inoculation with the fungus upon infestation with the insect significantly altered defence metabolism in resistant inbreds [11]. In sugarcane, Mahlanza et al. [12] showed that infection by *Fusarium* species, especially in lower internodes, affected resistance to a borer pest. *Fusarium* could also be entomopathogenic against *Sesamia* as it has been proved for other fungal endophytes [13]. In maize, the entomopathogenic performance of *Fusarium* has been studied with contradictory results: while *Fusarium* infection of the roots impacted negatively on western corn rootworm [14], higher pest densities were found in the stem and ear of field grown maize in *Fusarium* infected plants [15].

Carbon isotopes are considered a powerful tool for assessing time-integrated plant responses to environmental conditions. From the two naturally occurring carbon isotopes ^12^C and ^13^C, plants are able to discriminate against the heavier ^13^C isotope during gas exchange, as Rubisco preferentially fixes ^12^CO_2_ [16]. This isotope discrimination known as Δ ^13^C results in a lower ^13^C concentration in plants compared to the atmosphere. The carbon isotope signature (or composition) of a plant tissue (δ ^13^C) is a consequence of this discrimination during CO_2_ fixation. Theory has shown that δ ^13^C is strongly related to C_i_/C_a_, the leaf intercellular to atmospheric [CO_2_] ratio, as this ratio depends on both CO_2_ supply to the leaf intercellular spaces through stomata and the CO_2_ demand driven by the photosynthetic capacity of mesophyll. Thus, δ ^13^C is linked to A/E, the ratio of photosynthesis (A) to transpiration (E), and serves as an indicator of gas exchange traits such as carbon fixation, stomatal conductance, and transpiration [17, 18].

Both Δ ^13^C and δ ^13^C vary in response to a wide range of environmental factors, including abiotic and biotic stress. Additionally, variations exist among plant genotypes and between photosynthetic pathways [19]. Although both C3 and C4 plants are depleted in ^13^C compared to atmospheric CO_2_, their average δ ^13^C values range from − 20 to -35‰ for C3 plants and from − 11 to -15‰ for C4 plants [18]. In C3 plants, variations in Δ ^13^C and δ ^13^C are consistently linked to environmental conditions. Under drought, these traits have been used as indirect selection criteria for breeding water use efficient crop varieties [20, 21].

There are few studies on how biotic stresses affect plant δ ^13^C [22, 23]. Most available studies focus on boring insects, which induce isotopic enrichment in their hosts plants. This effect is likely due to water transport disruption, a process that mimics water stress [22, 23]. However, no studies have investigated the effects of *Sesamia*, or other stem borer pests, on δ ^13^C in maize. Similarly, no published reports were found on the impact of *Fusarium* infection, alone or in combination with these pests.

This study aimed to evaluate changes in maize composition (%C, δ ^13^C) induced by *Sesamia* infestation and to assess potential synergistic or antagonistic effects of simultaneous *Fusarium* colonization. Eight genotypes with contrasting responses to either biotic agent were selected for this purpose. The following hypotheses were tested: (a) *Sesamia* infestation will induce maize δ ^13^C changes similar to those caused by water stress; (b) variations in plant δ ^13^C will differ among inbreds and may be partially explained by their level of resistance to *Sesamia* and/or *Fusarium;* and (c) *Fusarium* seed infection may influence maize plants responses to subsequent infestation.

## Materials and methods

### Carbon analysis equipment and standards

Elemental and isotopic analyses of carbon were carried out using an elemental analyser (EA, Carlo Erba CNS 1508, Milano, Italy) coupled to an isotope ratio mass spectrometer (IRMS, Finnigan Mat, delta C, Bremen, Germany). Measurement accuracy was ensured through the use of elemental (LECO 502–694, 10.8 ± 0.26% C, Saint Joseph, MI, USA) and isotopic standards (IAEA-CH-6, sucrose, -10.4 ± 0.2‰; IAEA-CH-7, polyethylene, -31.8 ± 0.2‰; IAEA Vienna). Samples were weighted using a Sartorius MC5 micro-balance (Göttingen, Germany, accuracy ± 1 µg).

### Experimental protocol

Eight maize inbreds, either resistant (R+) or susceptible (R-) to *Fusarium* ear rot and *Sesamia* (thereafter *Fusarium*^*(R+)*^, *Fusarium*^*(R−)*^, *Sesamia*^*(R+)*^ and *Sesamia*^*(R−*^) were selected (Table 1).

**Table 1:** Inbreds lines used and their resistance levels to *Sesamia nonagrioides* and *Fusarium verticillioides*: R+, resistant; R-, susceptible [24–26]

Inbreds	*Sesamia nonagrioides* infestation	*Fusarium verticillioides* infection
PB130	R+	R-
EP77	R+	R+
A509	R+	R+
EP125	R+	R-
EP42	R-	R-
A637	R-	R+
A630	R-	R+
A239	R-	R-

**Fig. 1:**
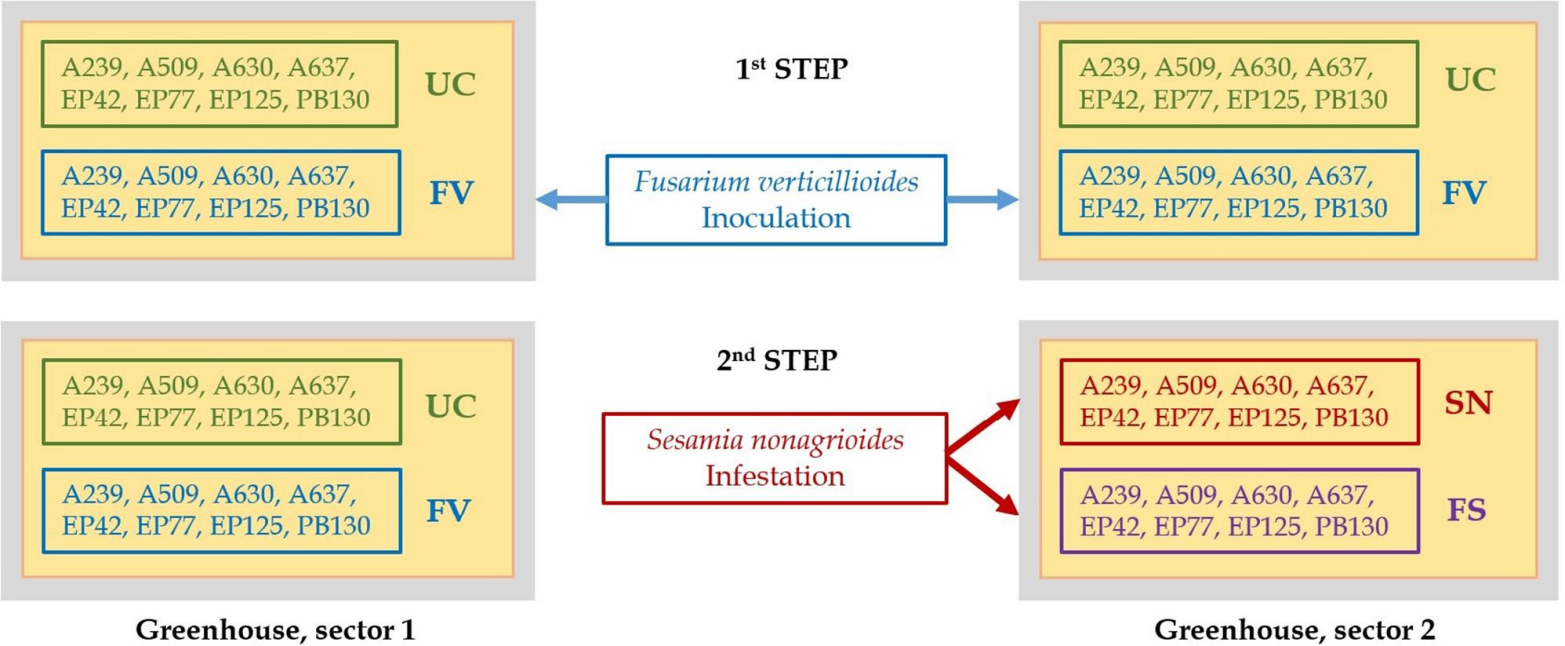
Schematic of the two-steps experimental protocol applied to the eight genotypes studied (A239, A509, A630, A637, EP42, EP77, EP125 and PB130) for obtaining the four planned treatments: UC, untreated control, no *Fusarium*, no *Sesamia*; FV, infected with *Fusarium verticillioides*; SN, infested with *Sesamia nonagrioides*; FS, infected with *F. verticillioides* and infested with *S. nonagrioides*

A total of 80 seeds per inbred were disinfected both internally and externally. Half of them were inoculated with *Fusarium* before being sown in 16 pots (2–3 seeds/pot), while the other half (non-inoculated) were sown in another 16 pots. The 32 pots were placed in a greenhouse. Once the plants were established, only one per pot was kept. For infestation, 8 inoculated and 8 non-inoculated plants per genotype were moved to an isolated greenhouse section and exposed to two *Sesamia* larvae per plant, generating four treatments: UC (untreated control, no *Fusarium*, no *Sesamia*), FV (*Fusarium*-inoculated), SN (*Sesamia*-infested), and FS (*Fusarium*-inoculated and *Sesamia-*infested) (Fig. 1).

Initially, six replicates per inbred-treatment were used, but due to germination issues only five replicates of SN and FS were available for inbreds A509, EP42 and A239. Inbred A637 showed poor germination in *Fusarium*-inoculated seeds, leading to the exclusion of FV treatment for this inbred. One month later, at the reproductive stage (with well-developed and lignified tissues), stem pith samples were collected from the first internode below the cob and lyophilized. Plants showing *Sesamia* damage were excluded from the control group, while non-damaged plants were excluded from the infested treatments (SN and FS).

### Elemental and isotopic analyses of maize

Maize subsamples (1.4 to 1.6 mg) were weighed into tin capsules (4 mm Ø x 6 mm), and analysed for their elemental (%C) and isotopic (natural abundance, δ ^13^C) composition. Isotopic composition was calculated using the following formula:

$${\delta ^{13}}C\, = \,\left( {{{{{({{{}^{13}C} \over {{}^{12}C}})}_{sample}}} \over {{{({{{}^{13}C} \over {{}^{12}C}})}_{PDB}}}} - 1} \right)\, \times \,{1000^{}}$$

Isotopic analysis followed several methodological principles, including some recommended by TD Jardine and RA Cunjak [27]. To ensure homogeneity of signal amplitudes between samples and standards, we adjusted their mass. Similarly, the signal from the internal reference injected at each analysis was harmonized by adjusting the flow rate from a CO_2_ pressure bottle calibrated against IAEA standards. Analytical accuracy and precision were verified using standards inserted after each series of samples. When wear on the EA combustion and reduction furnaces caused a deviation in accuracy of 0.3–0.4‰, they were replaced. If necessary, the isotopic values of samples were corrected according to the deviation between the measured and certified values of the IAEA standards used as reference in the analytical sequence. Finally, plants whose δ ^13^C deviated by more than 0.5‰ from the average of their respective treatments were subjected to a new analysis.

### Data analysis

An exploratory analysis of %C and δ ^13^C values was first carried out to identify any outliers or anomalies likely to affect the results. The normality of the data distribution was checked using Shapiro-Wilk’s W test, and the homogeneity of variance between groups was assessed by Levene’s test. If these assumptions were not met, the data were transformed using Tukey’s power scale. The data were then analysed using three rounds of two-way ANOVA, with treatment as the first factor, and either genotype, *Sesamia* resistance, or *Fusarium* resistance as the second factor (for %C and δ ^13^C). Significant differences between the group means were determined using the Bonferroni test for multiple comparisons, with a significance level set at *p* < 0.05. The proportion of the variance explained by each factor or interaction was estimated using the partial eta-squared coefficient (η_p_^2^). All statistical analyses were performed using the IBM-SPSS 28.0 software.

## Results

### ^13^C isotopic signature of stem piths

**Fig. 2:**
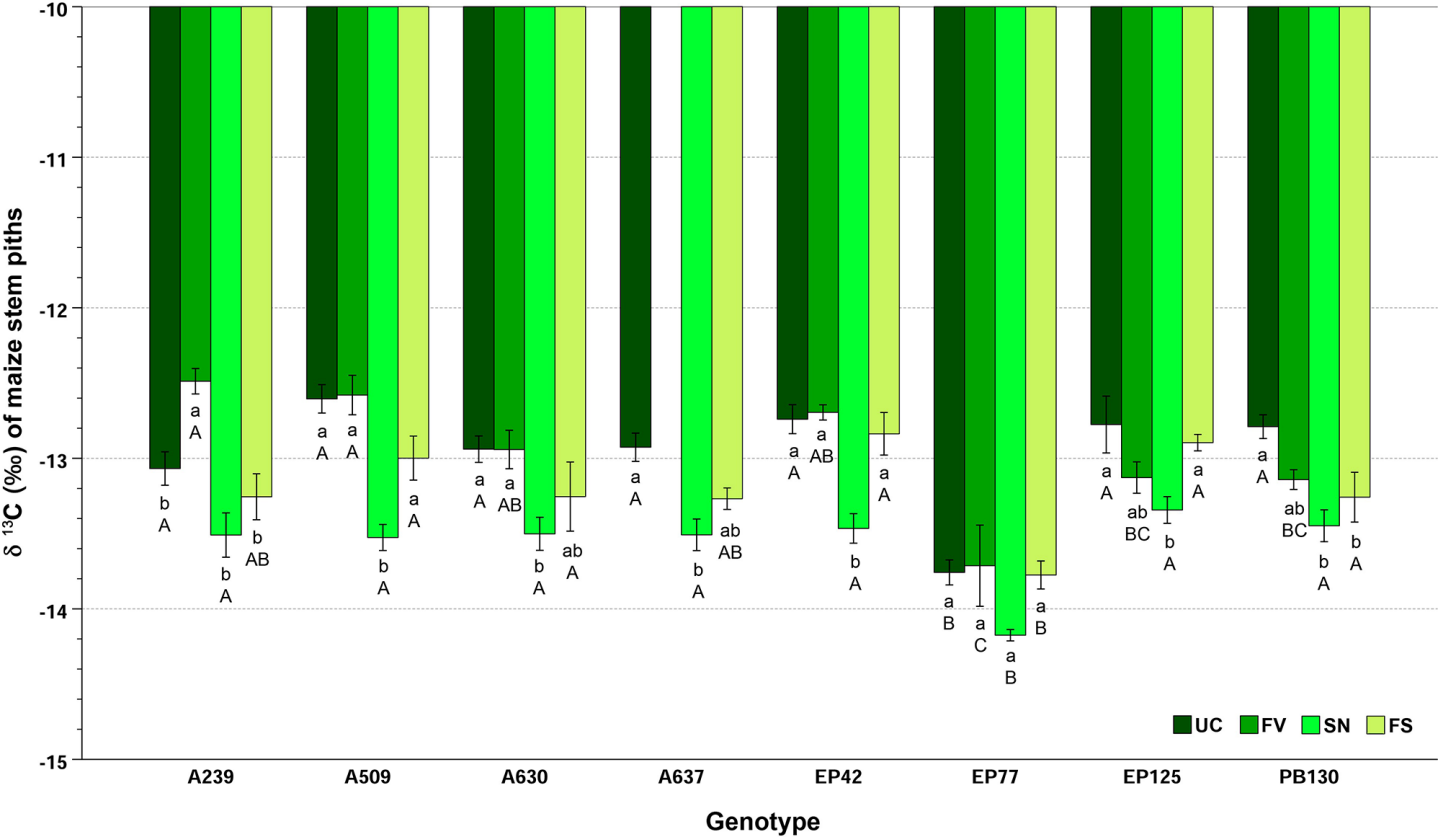
Mean ± standard error of δ ^13^C in maize stem piths for each genotype-treatment combination. Lowercase letters show differences (*p* < 0.05) between treatments within the same genotype; uppercase letters show differences (*p* < 0.05) between inbreds within the same treatment. Key of treatments: UC (untreated control, no *Fusarium verticillioides*, no *Sesamia nonagrioides*), FV (*Fusarium*-inoculated), SN (*Sesamia*-infested), and FS (*Fusarium*-inoculated and *Sesamia-*infested)

Two-way ANOVA revealed a significant influence of genotype (F = 21.63; *p* < 0.001), treatment (F = 45.30; *p* < 0.001) and their interaction (F = 1.95; *p* = 0.013) on the ^13^C isotopic signature in maize stem pith. These factors explained 51.8%, 49.1% and 21.7% of the total variance, respectively. Regarding the genotype effect, A509 had the highest (less negative) mean δ ^13^C value at -12.93‰, significantly different from most other lines, with the exception of EP42, EP125, and A239. Conversely, EP77 displayed the most negative value (-13.86‰), significantly lower than the other genotypes, while the remaining lines presented intermediate values without marked differences except for EP42, A637, and EP77. Regarding the treatment effect, the δ ^13^C isotopic signature followed a decreasing trend in the following order: FV ≈ UC (-12.95‰) > FS (-13.20‰) > SN (-13.56‰). The effects of the inbred and treatment factors were qualified by their significant interaction. In all treatments, EP77 displayed the most negative δ ^13^C with significant differences compared to the other lines, that did not differ among them except in a few pairwise comparisons for the FV treatment (A239 ≈ A509 > PB130 ≈ EP125; uppercase letters in Fig. 2). Specifically, A239 showed the lowest value in SN condition, but stood out for a significantly higher isotopic signature in FV condition than in the other treatments (Fig. 2). For the other genotypes, the lowest δ ^13^C value was recorded under SN condition, but the magnitude of significant differences with other treatments varied according to the line: A509 and EP42 (SN < UC, FV, FS), A630 (SN < UC, FV), EP125 (SN < UC, FS), A637 and PB130 (SN < UC), while for EP77 no significant differences were observed (Fig. 2).

**Fig. 3:**
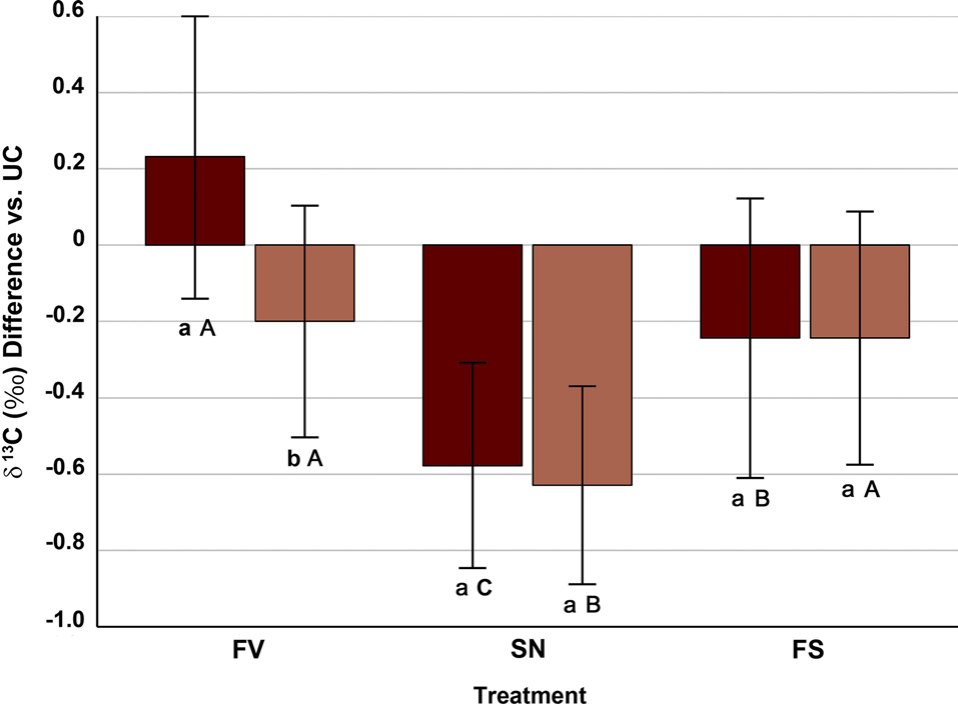
Mean ± standard error of the differences in δ ^13^C of maize stem piths between the infection/infestation treatments (FV, *Fusarium verticillioides*-inoculated; SN, *Sesamia nonagrioides-*infested; FS, *Fusarium*-inoculated and *Sesamia-*infested) and the untreated control (UC, no *Fusarium*, no *Sesamia*). Within a given treatment, different lowercase letters show differences (*p* < 0.05) between inbreds resistant (light boxes) and susceptible (dark boxes) to *S. nonagrioides*. Within an inbred group, different uppercase letters show differences (*p* < 0.05) between treatments

The two-way ANOVA with resistance to *Sesamia* and treatment as main sources of variation showed that both factors (F = 8.40; *p* = 0.004 and F = 41.41; *p* < 0.001, respectively) and its interaction (F = 5.85; *p* < 0.001) affect significantly the differences in ^13^C isotopic signature of maize stem pith of FV, SN and FS treatments with regard to the double control UC (6.3%, 39.7% and 8.5% of the variance explained, respectively), SN treatment leading to a more intense decrease of δ ^13^C than FS and FV (Fig. 3).

**Fig. 4:**
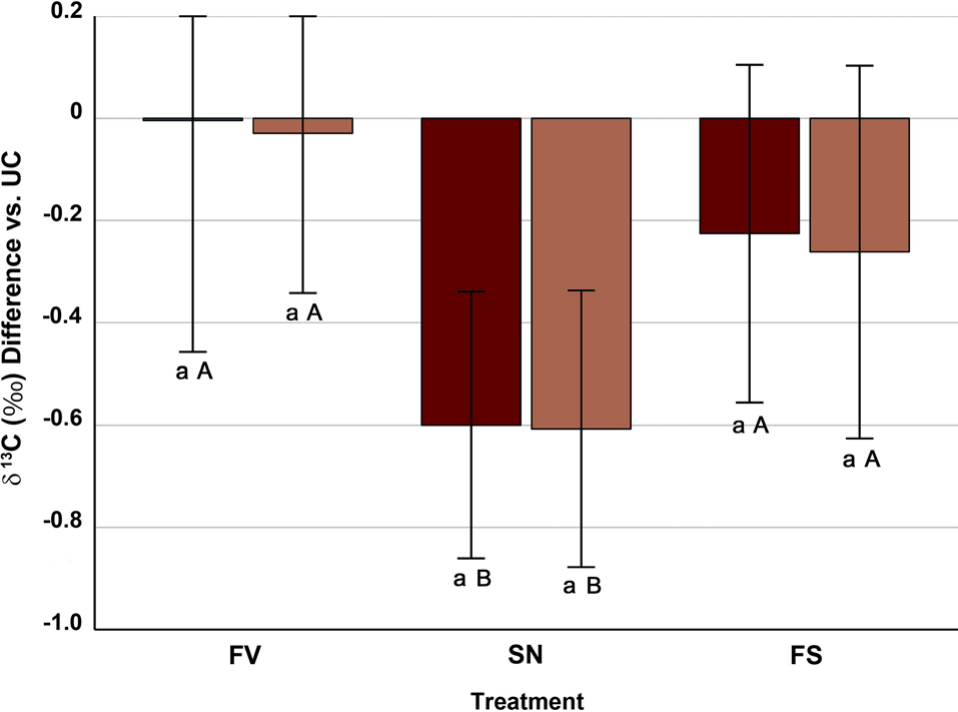
Mean ± standard error of the differences in δ ^13^C of maize stem piths between the infection/infestation treatments (FV, *Fusarium verticillioides*-inoculated; SN, *Sesamia nonagrioides-*infested; FS, *Fusarium*-inoculated and *Sesamia-*infested) and the untreated control (UC, no *Fusarium*, no *Sesamia*). Within a given treatment, different lowercase letters show differences (*p* < 0.05) between inbreds resistant (light boxes) and susceptible (dark boxes) to *F. verticillioides*. Within an inbred group, different uppercase letters show differences (*p* < 0.05) between treatments

The two-way ANOVA with resistance to *Fusarium* and treatment as main sources of variation showed that only the latter (F = 32.68; *p* < 0.001) had a significant effect on ^13^C isotopic response to *Fusarium*, *Sesamia* and the double treatment FS (*p* < 0.001; 34.2% of the variance explained). Compared with UC, the FV treatment did not modify consistently the δ ^13^C, whereas the FS and, especially, the SN treatments led to a decrease of δ ^13^C, which was similar in *Fusarium*^*(R−)*^ and *Fusarium*^*(R+)*^ (Fig. 4).

### C content of stem piths

**Fig. 5:**
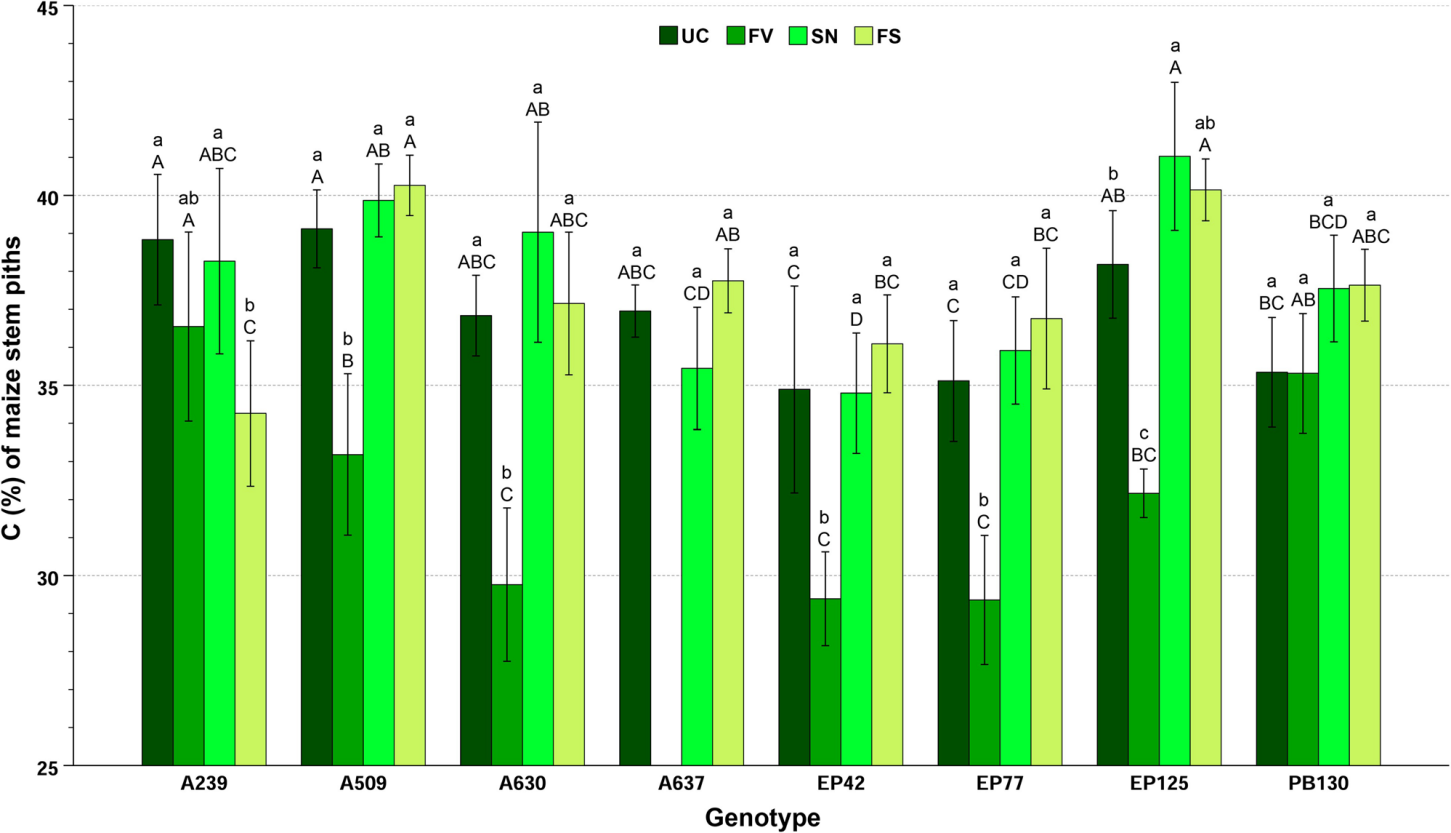
Mean ± standard error of C content (%) in maize stem piths for each genotype-treatment combination. Lowercase letters show differences (*p* < 0.05) between treatments within the same genotype; uppercase letters show differences (*p* < 0.05) between inbreds within the same treatment. Key of treatments: UC (untreated control, no *Fusarium verticillioides*, no *Sesamia nonagrioides*), FV (*Fusarium*-inoculated), SN (*Sesamia*-infested), and FS (*Fusarium*-inoculated and *Sesamia-*infested)

The C content of maize stem pith was significantly influenced by the inbred (F = 18.78; *p* < 0.001) and treatment factors (F = 89.14; *p* < 0.001) and their interaction (F = 6.02; *p* < 0.001) that explained 47.9%, 65.2% and 45.7% of the total variance, respectively. EP125, A509 and A239 presented the highest C contents (37–39%) and EP42 and EP77 the lowest (34–35%), but the significant differences among inbreds were scarce: EP125 > A637, PB130, A630, EP77, EP42; A509 > A630, EP77, EP42; A239 > EP77, EP42; and EP42 < than the rest, except EP77. Treatment FV (around 32% C) significantly differed from the other treatments (37–38% C). Although the inbred x treatment interaction was significant, the effect of the treatment was similar for all inbreds, except for A239 (Fig. 5).

**Fig. 6:**
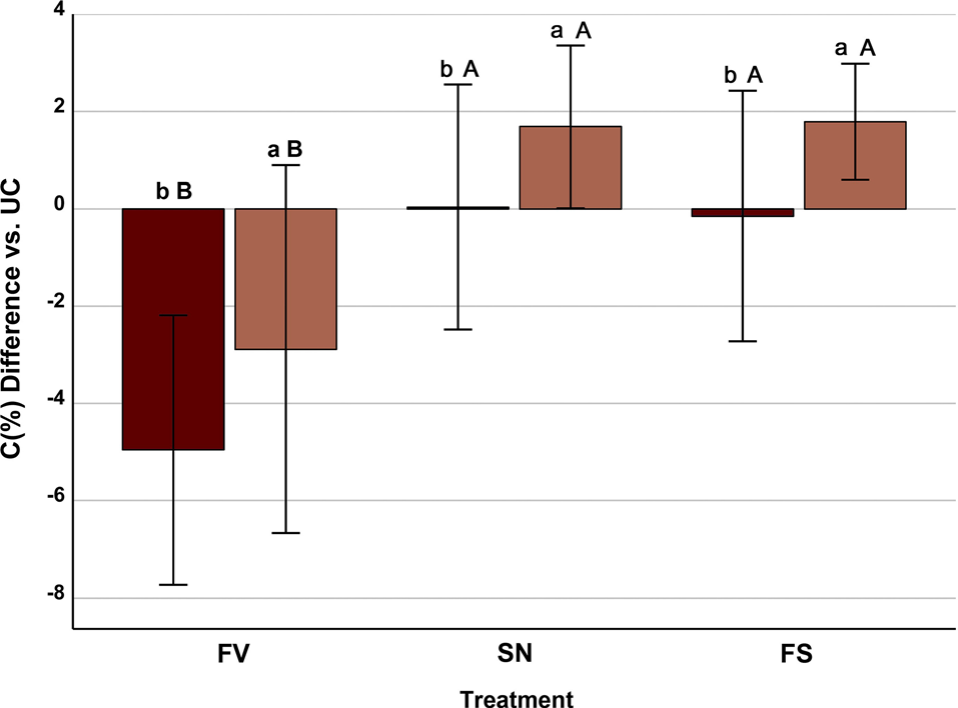
Mean ± standard error of the differences in C content (%) of maize stem piths between the infection/infestation treatments (FV, *Fusarium verticillioides*-inoculated; SN, *Sesamia nonagrioides-*infested; FS, *Fusarium*-inoculated and *Sesamia-*infested) and the untreated control (UC, no *Fusarium*, no *Sesamia*). Within a given treatment, different lowercase letters show differences (*p* < 0.05) between inbreds resistant (light boxes) and susceptible (dark boxes) to *S. nonagrioides*. Within an inbred group, different uppercase letters show differences (*p* < 0.05) between treatments

The two-way ANOVA considering resistance to *Sesamia* and treatment as main sources of variation showed that both factors (F = 14.74; *p* < 0.001; and F = 37.80; *p* < 0.001) affect the responses in C content of FV, SN and FS treatments (10.5 and 37.5% of the variance explained, respectively). In all treatments (FV, SN and FS), the C content was higher for *Sesamia*^*(R+)*^ than for *Sesamia*^*(R−)*^ inbreds (Fig. 6).

**Fig. 7:**
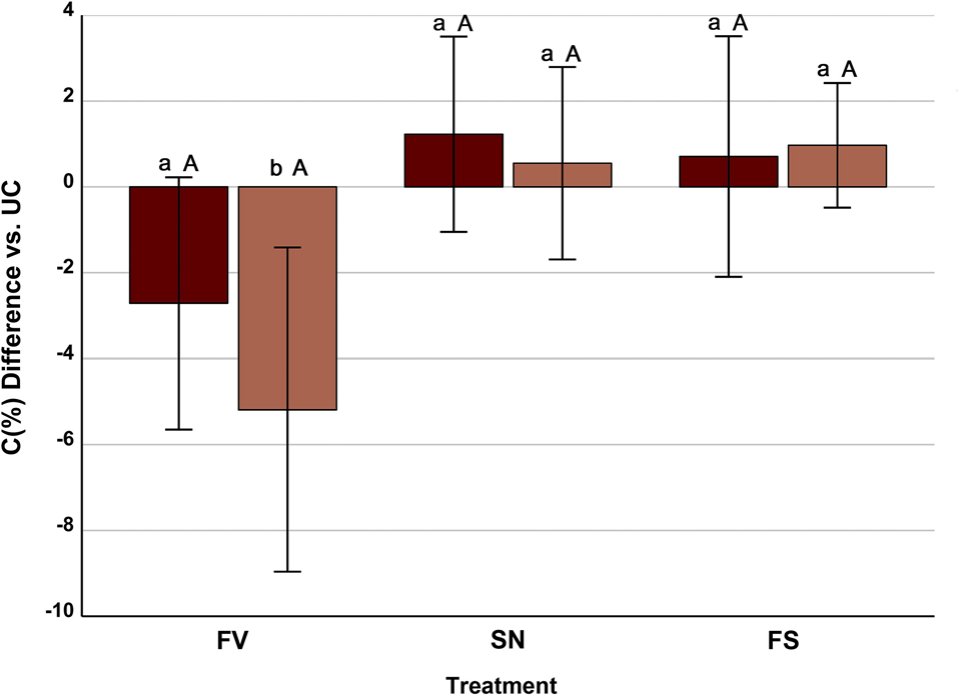
Mean ± standard error of the differences in C content (%) of maize stem piths between the infection/infestation treatments (FV, *Fusarium verticillioides*-inoculated; SN, *Sesamia nonagrioides-*infested; FS, *Fusarium*-inoculated and *Sesamia-*infested) and the untreated control (UC, no *Fusarium*, no *Sesamia*). Within a given treatment, different lowercase letters show differences (*p* < 0.05) between inbreds resistant (light boxes) and susceptible (dark boxes) to *F. verticillioides*. Within an inbred group, different uppercase letters show differences (*p* < 0.05) between treatments

The two-way ANOVA with resistance to *Fusarium* and treatment showed that only the interaction between both factors (F = 4.05; *p* = 0.022; 10.8% of the variance explained) affect the differences among responses in C content to the FV, SN and FS treatments, the only significant difference among responses being that between susceptible and resistant genotypes to FV treatment (Fig. 7).

## Discussion

It must be highlighted that the SN treatment always had the most negative δ ^13^C values, being significant the differences in 6 out 8 genotypes irrespectively of their resistance to *Sesamia*. Compared to the mean δ ^13^C of UC plants (-12.95‰), SN plants showed a decrease around − 0.6‰. This result is opposed to expectations of decreased ∆ ^13^C and increased δ ^13^C based on results obtained in C3 plants attacked by borer insects [22, 23]. However, one of the main consequences of maize stem boring by *Sesamia* is the water stress caused by larval galleries [28]. The active tunnelling behaviour of corn borers feeding on the stalk pith also damages the surrounding vascular tissues. This results in a partially disrupted transport of water and essential nutrients throughout the plant, causing drought-like symptoms in adult maize plants. Because of that, maize δ ^13^C changes caused by *Sesamia* attack could be similar to those caused by drought stress. Thus, we would expect an increase in Δ ^13^C and a decrease in δ ^13^C, as in the few reports on the relationship between drought stress and isotopic discrimination in maize [21, 29, 30]. These results, opposed to the relationships found in C3 plants, are attributed to the particularities of the CO_2_ fixation process in C_4_ plants like maize [21].

Under seed inoculation with *Fusarium* alone (no infestation), the δ ^13^C increased in *Sesamia*^*(R−*)^ inbreds and decreased in *Sesamia*^*(R+)*^, with the resistant inbreds EP77, EP125 and PB130 showing the lowest levels for δ ^13^C. Maize infection by *Fusarium* has been associated to increased or decreased stomatal conductance, and discrepancies among studies could be attributed to the existence of genotype-dependent responses [31, 32]. Therefore, we hypothesize that the less negative δ ^13^C of most *Sesamia*^*(R−*)^ inbreds under systemic colonization with *Fusarium* could be associated to enhanced tolerance to xylem disruption mediated by lowering stomatal conductance; meanwhile, other inbreds would increase stomatal conductance.

Seed infection by *Fusarium* significantly reduced the percentage of carbon in the stem pith and there are at least two possible explanations, not mutually exclusive: (a) the fungal biomass itself because the C/N ratio of *Fusarium* sp. is almost five times lower (28 ± 7 vs. 146 ± 1) than that of maize stalks [33, 34]; and (b) decreased maize cell wall lignification, as the C content of lignin is lower than that of cellulose [35]. However, although lignin is ^13^C depleted compared to other plant constituents [36, 37], the δ ^13^C in in stem piths of FV and UC plants were similar. Besides, no significant difference was found between the Klason lignin content in the stem pith of plants developed from *Fusarium*-inoculated and *Fusarium*-free seeds [11]. Therefore, the possible role of reduction of cell wall lignification on the observed carbon changes should be disregarded.

Under infestation with *Sesamia*, the presence of *Fusarium* in the seed reduced δ ^13^C half compared to reduction caused by infestation alone. This result suggests that seed infection by *Fusarium* would interfere in the photosynthesis response of maize to damage by stem borers and could, simultaneously, interfere in the defensive response [38]. Nitrogen content depletion and C/N enrichment are indicators of induced defence against insect attack [39–43]. Agreeing with the expectations, after *Sesamia* infestation these indicators varied less in plants primed by seed inoculation than in non-inoculated ones [11]. Carbon content increments in response to *Sesamia* treatments (SN and FS) were always higher in resistant than susceptible genotypes. This result could be consequence of C-expensive defence enrichment in some resistant inbreds, such as A509 and EP125 that presented the highest levels for C content after both treatments [44].

As shown before, resistant inbreds presented the highest levels for C content after infestation (A509 and EP125) and/or the lowest δ ^13^C after FV treatment (EP77, EP125 and PB130). Therefore, neither single parameter could be used as proxy of resistance to *Sesamia* because each one would be related to a different defence mechanism and the inbreds used in the current study did not share the same mechanism of defence [45, 46].

## Conclusions

Maize stem infestation by *Sesamia* led to a substantial stem pith δ ^13^C decrease of 0.6‰, causing drought-like symptoms. This reduction was partially counterbalanced in plants primed by *Fusarium* infection, in which the defensive response seemed diminished compared to *Fusarium*-free-seed plants. Unfortunately, neither the C content nor the ^13^C isotopic fingerprint could be used as proxy of resistance to *Sesamia* in breeding programs. Nevertheless, the ^13^C isotopic and C content signatures provide relevant information about maize defence mechanisms.

## Data Availability

The data that support the findings of this study are available from the corresponding author upon reasonable request.
